# Financial Incentives Alone Versus Incentivized Partner Support for Promoting Smoking Cessation During Pregnancy and Postpartum: Protocol for a Non-Randomized Single-Blinded Study

**DOI:** 10.2196/resprot.7907

**Published:** 2017-10-31

**Authors:** Mai Frandsen, Megan Thow, Stuart G Ferguson

**Affiliations:** ^1^ School of Health Science Faculty of Health University of Tasmania Launceston Australia; ^2^ School of Medicine Faculty of Health University of Tasmania Hobart Australia

**Keywords:** smoking, pregnancy, financial incentives, contingency management, partner support

## Abstract

**Background:**

Smoking tobacco remains the most significant modifiable cause of adverse pregnancy outcomes and contributor to ongoing maternal and infant ill-health. Pregnancy for many is a time of heightened health focus, with the primary motivation being the well-being of the unborn child. Yet, many women continue to smoke throughout their pregnancy. Despite this heightened motivation and known health risks, interventions to date have not effectively curbed the rate of smoking during pregnancy and they remain as high as rates among the general population. One promising strategy has been to incentivize these women to quit. However, incentives-based studies have not shown or reported long-term efficacy. Here, we present the protocol of a trial exploring the effect of incentivized partner support on pre- and postpartum smoking cessation.

**Objective:**

The aim of this study is to determine whether providing incentives to both the expectant mother and her support person in promoting short- and long-term smoking cessation during pregnancy is more effective than incentives to the expectant mother alone.

**Methods:**

This protocol is designed as a non-randomized, single-blinded trial to determine the efficacy of incentivized partner support, compared to participant incentive only, in promoting smoking cessation during pregnancy and postpartum. All eligible pregnant women receiving antenatal care via the Tasmanian Health Service (Australia) will be invited to participate. Participants will be eligible for monthly quit-contingent shopping vouchers if they verify, via carbon monoxide breath sample, as being abstinent from smoking. Participating women will be eligible for vouchers until 6-months postpartum and will be followed up at 12-months postpartum.

**Results:**

The recruitment phase of this study has concluded. Results are expected to be published by the end of 2018.

**Conclusions:**

This study protocol extends the current literature on incentivized smoking cessation interventions for pregnant women by assessing the influence of incentivizing a support partner on short- and long-term abstinence. Key ethical considerations are discussed including potential for receipt (or not) of quit-contingent vouchers impacting negatively on the participant’s relationship with their partner. The findings of the study may have important implications for the role support partners are assigned in smoking cessation programs targeting pregnant women.

**Trial Registration:**

Australian New Zealand Clinical Trials Registry (ACTRN): 12615001158550; https://www.anzctr.org.au/ Trial/Registration/TrialReview.aspx?id=367981 (Archived by WebCite at http://www.webcitation.org/6tGKO28uh)

## Introduction

### Smoking and Perinatal Health

Smoking during pregnancy is recognized as the single most modifiable cause of poor pregnancy outcomes [1] and the risks to mother and baby have been reported extensively elsewhere [2]. Briefly, these risks include increased risk of miscarriage, preterm birth, low birth weight, major congenital abnormalities, and sudden infant death syndrome [2,3]. Less known long-term effects as a result of prenatal smoke exposure include increased risk of reduced neuromotor function, attention deficit hyperactivity disorder (ADHD), learning difficulties, and behavior dysregulation during childhood and adolescence [4-7]. Infant exposure to second-hand smoke has also been shown to further compound these negative health consequences [2]. Despite these known risks, many women continue to smoke during and after pregnancy, at rates comparable to the general smoking population [8,9]. Even when women manage to quit during pregnancy, most (up to 80%) relapse within 6 months of delivery [10]. Rates are considerably and consistently higher among certain already disadvantaged cohorts including young expectant mothers (in Australia, 34% of pregnant women 20 years or younger smoke) and women living in rural and remote areas (37% in Australia) [11]. More effective, targeted interventions are clearly needed for this high-risk group.

### Interventions for Promoting Cessation Among Pregnant Smokers

One of the most effective strategies for promoting smoking cessation among pregnant women is incentives-based interventions [12,13]. Providing incentives for pregnant smokers to quit has been shown to not only increase abstinence rates several-fold compared to any other type of treatment, but also to increase mean birth weight [12,14]. Further, in a review of the most common interventions (including cognitive behavior therapies, stages of change, feedback, pharmacotherapies, and other therapies), incentives-based programs were found to be the most cost-effective, producing a net cost benefit of US $3482 after factoring in intervention costs [12,15,16]. Incentives-based programs for pregnant smokers appear to promote successful postpartum abstinence rates (approximately 25% abstinent at 3 to 6 months postpartum), but since few studies have included long-term postpartum follow-up, the long-term efficacy is not well understood [17]. While one study reported a 12-month postpartum follow-up with promising cessation rates (0% to 44% depending on model of care), this study included participants who had quit within 1 month of enrolment, and thus the effectiveness of incentives-based interventions at promoting long-term abstinence remains unclear [18].

### Partner Support and Sustained Cessation

In a review of postpartum relapse prevention strategies, programs that involved the pregnant smoker’s partner were deemed necessary to maximize long-term cessation success [19]. To date, only one randomized controlled trial (RCT) has been conducted to examine the effect of partner support [20]. Participants were randomized to 3 groups: usual care, counseling support (6 counseling telephone calls; 3 during pregnancy, 3 postpartum), and counseling support combined with partner-support facilitated by a tailor-made “it takes two” booklet and video (partner was usually baby’s father who was also provided 6 separate counseling support telephone calls). No significant difference in participants’ smoking behavior was found between the groups. Interestingly, the authors found that positive support (behaviors characterized by cooperation and reinforcement of quitting behavior such as “compliment you on not smoking”) diminished linearly from baseline to 12 months postpartum and negative support (behaviors characterized by nagging and policing such as “comment on your lack of willpower”) decreased through pregnancy before increasing again postpartum.

The importance of the quality of support provided in partner support interventions has been found to be critical in promoting cessation among non-pregnant smokers [21]. In a study exploring the effect of positive and negative support behaviors on quit rates among female smokers, Cohen and Lichtenstein showed that women who reported receiving a higher ratio of positive supportive behavior compared to negative (using a shortened version of the Partner Interaction Questionnaire) from their spouse were more likely to quit. This emphasis on quality of support may explain why other studies found no effect for partner support [20-22].

Combining incentives programs with partner support, particularly the co-habiting and/or romantic partner (eg, expectant father) that emphasizes positive support, may therefore be an approach that fosters more effective long-term cessation for pregnant smokers. To our knowledge, only one study to date has explored incentivizing social support to promote smoking cessation in pregnant women. Donatelle and colleagues [23] compared 2 groups: 1 receiving usual antenatal care and 1 which included both the women and their chosen female non-smoking supporter (providing unstandardized, non-formalized, “natural” peer support only) provided with quit-contingent shopping vouchers. Participants in the incentivized partner support group were more likely to be quit at the end-of-pregnancy (32% versus 9%) and at the 2-month postpartum (21% versus 6%) time points compared to usual care. Since this study was not fully factorial (eg, intervention groups consisting of social support only and incentives only, not included), the effect of partner support over and above incentives could not be determined. Furthermore, the support person in the Donatelle et al [23] study was not the pregnant women’s spouse (eg, husband and/or father of child), but rather a female, non-smoking friend, who might arguably have had less vested interest in the health of the expectant child than the expectant father.

### Objectives

The purpose of this study is to determine whether providing incentives to both support person and expectant mother, if she is able to quit smoking, is more effective than providing incentives to expectant mother only, in promoting short- and long-term smoking cessation during pregnancy. In essence, the present study seeks to answer the question: “Can partners (eg, spouse) be incentivized to be more supportive and effective quit buddies to their pregnant smoking partners?”

Specifically, the study aims to determine whether (1) providing an incentive to both the support partner and expectant smoking mother to quit is more effective than providing an incentive to the pregnant smoker alone at promoting abstinence; (2) regardless of incentives, women who receive more positive cessation support from their partners, as measured by the Partner Interactive Questionnaire 20 (PIQ-20), are more likely to quit smoking; and (3) providing incentives for pregnant smokers to quit is more effective than “usual care” antenatal quit smoking services such as brief advice and referral to smoking cessation services (external telephone counseling and smoking cessation nurse) at promoting smoking cessation.

The primary outcome for the study is smoking status (ie, smoking or quit), as determined by self-report 7-day point prevalence, and carbon monoxide (CO) less than 7 particles per million (ppm) at the end-of-pregnancy time point. Secondary outcomes include effect of incentives on long-term (2 months and 12 months postpartum) abstinence and influence of the quality of partner support (positive compared to negative supportive behaviors) on smoking status.

## Methods

### Trial Design

This study will adopt a non-randomized, single-blinded, controlled 2-group (control and treatment) trial design.

### Participant Recruitment

All eligible pregnant women who smoke and live in Tasmania (Australia) are invited to participate in the study. Statewide recruitment is facilitated by drawing on data from the Tasmanian Health Service’s digital medical record (DMR) and encompasses multiple strategies to maximize reach. A research midwife, with access to the Tasmanian Health Service’s DMR, conducts “cold calling” of all women who self-reported smoking in the last 7 days during their initial antenatal “booking in” appointment (usually around 10 weeks gestation). Using information stored on the DMR, the midwife pre-screens women for eligibility (eg, self-report smoking, 16 years or older) and telephones them to invite them to participate in the study. With their verbal consent, the details of interested women are recorded and forwarded to the research team.

Antenatal staff of the Tasmanian Health Service (including physicians and midwives) provide study information (in the form of a flyer) to eligible participants during antenatal appointments. With their consent, the contact details of interested women are forwarded to the research team. In addition, the study is advertised through flyers placed in general practitioner (GP) clinics, outreach centers and community hubs across Tasmania. Informal advertising via social media (eg, Facebook) and television and radio interview exposure may also be utilized. Consequently, potential participants could also self-refer to the study by contacting the researchers directly.

### Eligibility Criteria

Women who express interest in participating in the study (either self-refer or consent to a health professional forwarding their contact details to research staff) are contacted by research staff and screened for eligibility using a previously validated protocol [24]. To be eligible for study entry, women must be (1) pregnant (20 weeks gestation or less); (2) at least 16 years of age; (3) self-report as being a current smoker (“even a single puff in the last 7 days”); (4) attending routine antenatal care provided by the Tasmania Health Service or participating GP center (Tasmanian, Australia); and (5) be able to attend a minimum of 3 appointments at 1 of the 3 data collection sites across Tasmania (Launceston General Hospital, Royal Hobart Hospital, or Mersey Community Hospital). Women younger than 18 years (but 16 years or older) require the consent of their parent, guardian, or senior antenatal health provider to participate in the study.

Potential participants are excluded from study entry if they self-report as non-smoking (ie, “have not smoked, even a single puff in last 7 days”) or if they have a cognitive or intellectual impairment that will inhibit fulfillment of participation requirements (eg, completion of surveys and/or attend organized study appointments). Participants who are found to be “gaming” (eg, those who tell researchers they are pregnant and/or smoking untruthfully to enroll in study) are also excluded from further participation and their data discarded. All participants are asked to nominate and are encouraged to bring along to study visits a support person, preferably a person they co-habit with (eg, spouse, father of child, or family member).

### Assignment of Interventions

Participants are assigned to the same group (either control or the treatment group) for 3 consecutive months (interchangeably for 18 months), such that participants recruited in the first 3 months are allocated to the control group and participants recruited in the following 3 months are allocated to the treatment group. This design was chosen to assist participant blinding to group allocation, given the heightened potential for the women recruited to know each other and/or discuss research participation. Since participants in this study are recruited following their first antenatal appointment, participants recruited around the same time will have similar due dates. As membership to parents’ groups and hospital-run parentcraft classes is usually assigned by due date/gestation, and due to the relatively small recruitment pool (Tasmania has a population of 515,000 and only 3 tertiary hospitals), adopting a fully randomized, parallel group controlled design would likely jeopardize participant allocation concealment (ie, differing incentive amounts), thus introducing bias (eg, recruited women declining/delaying enrolment due to allocation, participants not being as motivated to quit smoking due to lesser incentive amount). As well as assigning participants to the same group in 3-month intervals, participants who report knowing another participants or are recruited by another participant are assigned to the same group.

### Interventions

During their initial enrolment visit (visit 1), all participants and their support partners are offered a separate resource pack providing further information and quit references. The content of the packs, selected on the basis of existing resources developed and distributed by not-for-profit organizations and government-funded bodies (eg, Quitline, Cancer Council, and SIDS and Kids), includes informational brochures on the topics of smoking and pregnancy, quitting smoking during pregnancy, a guide for quitting smoking, and smoking and sudden infant death syndrome. A referral and resources summary list (eg, further websites and available mobile phone apps) is also provided that includes telephone numbers of local counseling services. The resource pack specifically designed for partners contains information about the effect of second-hand smoke on children, quit smoking products, and how to be an effective quit buddy. Following the single-blinded, consecutive-month schedule, women are allocated to either the control or treatment group during their enrolment session (Textbox 1).

**Control and Treatment group incentives.**

Group

- Control
  - Only the participant is rewarded with a AUD $50 voucher if they verify as quit during monthly visits
- Treatment
  - Both participant (1 x AUD $50) and their designated support person (1 x AUD $50) receive a shopping voucher if the participant verifies as quit during monthly visits
  - If partner is not present during study visits, participant collects both vouchers

All participants are encouraged to make a quit attempt in the 2 weeks following the enrolment visit in order to promote quit status (verified by less than 7 ppm CO breath sample) and enable incentive issue at the first monthly follow-up, although it is emphasized that each participant is able to decide their own quit timeframe and approach. No other formal smoking cessation counseling support is provided.

All participants are asked to attend 2 further study visits—visit 2 (end-of-pregnancy) and visit 3 (end-of-study)—and have the opportunity to attend monthly visits to verify their non-smoking status and receive incentive voucher/s.

### Quit-Contingent Incentive Vouchers and Study Compensation

All participants attending a scheduled visit are provided with some form of voucher compensation. For participants who are still smoking, an AUD $10 voucher is offered. Participants who verify as quit are offered the shopping voucher amount consistent with their group allocation. If a participant is not abstinent at any visit during pregnancy, they are still eligible to receive the quit-contingent incentive at any subsequent visit during pregnancy if they provide a CO reading of less than 7 ppm.

### Verification of Smoking Status

During each visit and telephone call, participants are asked if they have smoked, even a single puff, in the last 7 days. During study visits, participants complete a 14-day timeline follow-back questionnaire as an assessment of self-reported smoking. To verify self-report, all participants are required to provide 2 expired air CO samples using a piCO Simple Smokerlyzer [25]. Expired CO was chosen as the most appropriate biochemical verification method in this study as it is not sensitive to the use of nicotine-containing medication (such as nicotine replacement therapy which may be used by participants), is non-intrusive, immediate (facilitates immediate provision of quit-contingent incentive vouchers), and inexpensive. The CO readings are recorded and if the average of the 2 samples is less than 7 ppm, the participant’s smoking status is recorded as quit and she receives a voucher incentive. A cut-off value of less than 7 ppm was selected based on recommendations from the National Institute of Health and Care Excellence [15], in addition to the precedent set by existing research in the field which accounts for likely second-hand smoke exposure (eg, from support partner) [18,26]. To check whether our results are sensitive to the CO cut-off value, we also repeat our primary analysis using a CO level of less 4 ppm as the cut-off for abstinence. Substantial discrepancies between the results obtained with the 2 cut-off values suggests that participants are “gaming” the system (that is, continuing to smoke but cutting down just enough to receive the abstinence reimbursement). Based on results of previous similar studies [27], we do not expect “gaming” among participants for incentives to be a significant issue.

### Procedure

#### Study Visits and Follow-Up Calls

A schematic diagram summary of the time-schedule of visits, rewards, and data collection at each visit is shown in Multimedia Appendix 1.

#### Enrolment Visit

During the enrolment visit (visit 1) participants are asked to complete a battery of questionnaires and provide 2 CO breath samples. Regardless of group allocation, participants are asked to nominate a support person (they are asked to have this person in mind when completing the Partner Interaction Questionnaire), preferably the person they are living with (eg, partner or family member), whose contact details are recorded along with the participant’s own details to assist with future correspondence. While no smoking cessation counseling is provided, participants are offered a resource pack for themselves and their support partner.

#### End-of-Pregnancy Visit

Regardless of smoking status, all participants are required to attend an end-of-pregnancy study visit. This second visit occurs at approximately 8 months gestation and participants are asked to provide 2 CO samples and complete a series of questionnaires.

#### End-of-Study Visit

Regardless of smoking status, all participants are required to attend an end-of-study visit, which occurs at approximately 2 months postpartum. Participants are asked to provide 2 CO samples and complete a series of questionnaires including the End-of-Study Questionnaire.

#### Monthly Telephone Calls and Verification Visits

Monthly telephone calls are conducted with all participants to determine smoking status. During the phone calls, participants are asked “Have you had a cigarette (even a puff) in the past 7 days?” If a participant self-reports as abstinent, they are invited to attend a follow-up verification visit to verify their smoking status (and receive a voucher incentive), which is booked at their next convenience. If the participant self-reports smoking in the last 7 days during the monthly telephone call, they are advised that they are not eligible for the incentive that month and will be contacted again the following month. This monthly payment schedule limits the additional participation burden on participants, and where possible, is scheduled on days when they are attending routine hospital antenatal appointments. This monthly payment schedule was also utilized by the only other study published using incentivized partner support [23]. Participants who verify as abstinent at their end-of-study visit (visit 3) are eligible to receive monthly calls and incentives until 6 months postpartum. Participants who are still smoking at the end-of-study visit no longer receive monthly calls (and are ineligible for any further incentives) from that date. Multiple (up to 5) attempts are made to contact each participant for their scheduled monthly telephone calls and visits. Text messages (short message service, SMS) are also utilized to try to contact those difficult to reach. However, the study adopts an intention-to-treat approach such that if participants become un-contactable at any time throughout the study, it is presumed they are smoking [28].

#### 12-Month Telephone Call and Visit

Each participant, regardless of smoking status, is contacted via telephone 12 months after the delivery of their baby. The 12-Month Follow-Up Questionnaire is administered via telephone and participants are asked their current smoking status. Participants who self-report abstinence (7-day point prevalence) during this call are invited to verify smoking abstinence via a CO breath sample in a follow-up visit and be offered an AUD$10 shopping voucher (regardless of verification status) to thank them for their participation. This marks the completion of their participation in the study.

### Study Questionnaires

A battery of questionnaires are issued to participants during study visits.

#### Baseline Questionnaire

The Baseline Questionnaire was developed to obtain demographic information (eg, age, highest level of education, income), information about smoking characteristics (eg, smoking status, cigarettes per day, dependence [Fägerstrom Test for Cigarette Dependence [29]], smoking history, and partner’s smoking characteristics), and pregnancy (eg, gestation, gravidity, smoking during previous pregnancies). This questionnaire is administered only once at study enrolment (visit 1).

#### Smoking Knowledge Quiz

The Smoking Knowledge Quiz was developed from existing publicly available and scientifically reliable smoking information and is similar to the questionnaire used in a previous study [30]. The quiz requires participants to answer questions regarding their knowledge of the safety and effectiveness of smoking cessation treatments and techniques during pregnancy, as well as the health risks associated with cigarette smoking for themselves and their baby. Participants’ responses to this quiz are reviewed with the participant at the time of the enrolment visit to prompt discussion of health risks and available cessation aids. The quiz is administered once only at study enrolment (visit 1).

#### Smoking Status Questionnaire

The Smoking Status Questionnaire is a self-report, 14-day cigarette timeline follow-back which requires participants to report the number of cigarettes smoked each day for the previous 2-week period [31]. This questionnaire is completed at each study visit.

#### Reasons for Quitting Questionnaire

The Reasons for Quitting Questionnaire (RFQ) is a 24-item questionnaire measuring an individual’s motivation for quitting [32]. Reponses to items such as “I want to quit smoking because I am concerned that I will suffer from a serious illness if I don't quit” are scored on a 5-point Likert scale from 0 (not at all true), to 4 (extremely true). This questionnaire is completed at each study visit.

#### Partner Interaction Questionnaire

The Partner Interaction Questionnaire (PIQ) is used to gather information about the level of partner support [33]. It includes 10 positive (PIQ-POS) (eg, “Express pleasure at your efforts to quit”) and 10 negative (PIQ-NEG) (eg, “Comment on your lack of willpower”) partner behaviors that could be expressed towards a quitting partner. This questionnaire is completed at each study visit.

#### End-Of-Study Questionnaire

The End-of-Study Questionnaire was developed to collect information after participants have had their baby and is conducted around 2 months postpartum during the scheduled end-of-study visit (visit 3). This questionnaire includes questions about smoking status, cigarettes per day, experience of study participation and receiving incentives, treatments/methods used to aid quit attempt, partner support, and partner smoking status, as well as any birth complications experienced.

#### 12-Month Follow-Up Questionnaire

The 12-Month Follow-Up Questionnaire is administered via telephone during the 12-month follow-up telephone interview. This marks the completion of study participation. Participants are asked to describe their smoking status, experience of being in the study, details of partner support of quit attempts, and their overall general well-being and that of their baby. If participants are quit, they are invited to return to the study center to verify.

Fetal growth and birth outcomes are also collected from babies of consenting participants (a separate consent form is provided seeking access to this information) including head circumference, femur length, heart/vascular data, birth weight, Apgar score (overall health at birth), gestational age, and digital medical records of neonatal intensive care unit (NICU) or respiratory admissions (if applicable). These data will be used for exploratory analyses to determine any associations with intervention effects.

### Sample Size and Checks

The study is powered on the primary hypothesis (H1) which requires a sample size of 108 (see below). This is consistent with a review of previous research in the area, which revealed sample sizes ranging between 40 and 220 with approximately equal group sizes [17]. To ensure particular features of the study protocol do not influence the data, checks will be conducted during the analysis phase to control for influence of different antenatal service sites being used for recruitment and testing, as well as any cyclical effects of the cyclical monthly recruitment design.

### Planned Analyses

Analyses are planned to test each of the 4 hypotheses.

#### Hypothesis 1

The first hypothesis (H1) is that providing an incentive to both the support partner and expectant smoking mother to quit is more effective than providing an incentive to the pregnant smoker alone at promoting abstinence during pregnancy.

The quit rates of the treatment and control groups will be compared at the end-of-pregnancy time point (visit 2) using chi-square analysis. While no study to our knowledge has compared participant-only incentive with combined participant and support partner incentive, Donatelle et al [23] compared a usual care control group with an incentive plus partner incentive treatment group and found that at the end-of-pregnancy, 32% of treatment group participants had quit (n=105) compared with 9% in the control group (n=102), which indicates a large effect size (*χ*^2^=18.4, N=207, *d*=0.62) [23]. Since the control group in the present study is also receiving incentives, it is anticipated that the effect size may be moderate, rather than large. As such, to determine if there is a significant difference in proportion of women who have quit between control group and treatment group, with the power of .80 to detect an effect size of 0.30, the study will require a total sample size of 108.

#### Hypothesis 2

The second hypothesis (H2) is providing an incentive to both the support partner and expectant smoking mother to quit is more effective than providing an incentive to the pregnant smoker alone at promoting abstinence in the postpartum period.

As per H1 above, treatment and control group abstinence rates will also be compared at the end-of-study (visit 3) and at 12-months postpartum follow-up time points to determine the effect of incentivized partner support on postpartum abstinence. A multi-comparison adjustment will be applied to control for family-wise error.

#### Hypothesis 3

The third hypothesis (H3) is regardless of incentives, women who receive more positive cessation support from their partners (as measured by PIQ-20) are more likely to quit smoking.

Participants, regardless of group allocation, will be regrouped to either PIQ-POS or PIQ-NEG, as determined by the ratio of positive compared to negative support they report receiving from their support partners. The abstinence rates of these regrouped PIQ-POS and PIQ-NEG participants will then be compared at 3 time points; end-of-pregnancy, end-of-study (2 months postpartum), and 12 months postpartum.

#### Hypothesis 4

The fourth hypothesis (H4) is providing incentives for pregnant smokers to quit is more effective than “usual care” antenatal smoking cessation support at promoting smoking cessation.

The quit rates of participating women will be compared to the average quit rate of a historical control of women receiving antenatal care by the Tasmanian Health Service from 2011 to 2015 [34].

## Results

The recruitment phase of the study has concluded and postpartum data collection is ongoing. Data collection is anticipated to be complete by late 2017. Outcomes of the trial will be published within a year (12 months) of completing final 12-month follow-up data collection. The study results will be disseminated via conference presentations and papers published in academic peer-reviewed journals. The participants, healthcare professionals, and public will be informed of the study results through email correspondence and local media.

## Discussion

The project has been reviewed and approved by the Tasmanian Health and Medical Research Ethics Committee (H0014568). Prior to enrolling in the study, all participants provide verbal and written consent. Furthermore, additional checks are conducted throughout the study to ensure participant safety in light of details receiving particular attention during the ethical review process. The first is the risk of a negative impact of the receipt (or not) of quit-contingent shopping vouchers on the study participant’s relationship with their partner, with the concern that this may be a potential trigger for family violence or other threat to the safety of the woman (and/or unborn child). During each study visit or telephone call, participants are asked if study participation is impacting negatively on their relationship with their partner. If real or perceived risk is present, the study research officer ensures the safety of the participant by making urgent contact with and referral to the antenatal Social Work service (or after-hours emergency/crisis social work service) at the relevant public hospital to arrange safe accommodation and other services as indicated. The second is the risk of potential distress should a participant experience adverse pregnancy or other outcomes (eg, fetal or infant death) while involved in the study. The research midwife conducts monthly checks of participants’ pregnancy and health status via the Tasmanian Health Service’s digital medical records. In these rare occasions, no further contact is initiated with the participant and her involvement and data is withdrawn. However, should the participant request to continue to participate, she is welcome to do so.

## Appendix

Multimedia Appendix 1 Summary of the time-schedule of visits, rewards, and data collection.

## Abbreviations

AUD: Australian Dollars
CO: Carbon monoxide
DMR: digital medical record
GP: general practitioner
PIQ: Partner Interaction Questionnaire
PIQ-NEG: PIQ negative
PIQ-POS: PIQ positive
ppm: particles per million
