# Supplementary material for: Financial Incentives Alone Versus Incentivized Partner Support for Promoting Smoking Cessation During Pregnancy and Postpartum: Protocol for a Non-Randomized Single-Blinded Study
Source: JMIR Res Protoc. 2017 Oct 31;6(10):e209. doi: 10.2196/resprot.7907 (PMC5686414; doi:10.2196/resprot.7907)
Supplement: Multimedia Appendix 1 [file resprot_v6i10e209_app1.pdf]

UNIVERSITY of TASMANIA | DIVISION OF RESEARCH

17 December 2014

Dr Mai Frandsen
School of Health Science
Locked Bag 1320
LAUNCESTON TAS 7250

Dear Mai

University of Tasmania – Research Enhancement Grants Scheme (REGS) - 2015

I am pleased to advise that your REGS application for "SF Emersion Study (Smoke Free Expectant Mothers through Incentivized Partner Support)" has been successful. Funding awarded under this grant is $18,246.

The conditions of the grant are as follows:

- The term of the grant is one year commencing 1 January 2015.
- Grant funds must be spent as outlined in the application by the project close date of 31 December 2015. No extension will be approved. Unspent funds will be returned to central funding.
- A Final Report, on the template available on the Research website, must be submitted to the Director, Research Services by 31 March 2016.
- Funds must not be used for conference attendance. Conference Central Support funding is available for this purpose.
- Grants are for internal purposes only. No portion of the funding can be transferred to other Universities or collaborating organisations (including industry partners).
- If a sole CI leaves the employment of The University of Tasmania then the grant must be relinquished.
- Any equipment that is purchased with this funding remains the property of the University of Tasmania.
- Funding cannot be used for CI salaries, teaching relief, or to fund a Research Higher Degree Candidate's stipend or fees.
- You will submit an ethics application for the project (if required).

Please complete the Acceptance Offer Form (attached) and return it to your Funding Officer **Che O'May** at Che.OMay@utas.edu.au. Please retain a copy for your records. Your Funding Officer will then open an account for your project, pending ethics approval (if required).

I wish you well with your project and look forward to hearing more about your achievements in your Final Report.

Yours sincerely

Professor Paddy Nixon
Deputy Vice-Chancellor (Research)
